# “Only My Husband and My Doctor Know. And You, Girls”: Online Discussions of Stigma Coping Strategies for Russian Surrogate Mothers

**DOI:** 10.3390/ijerph182111325

**Published:** 2021-10-28

**Authors:** Daphna Yeshua-Katz, Natalia Khvorostianov

**Affiliations:** Department of Communication Studies, Ben-Gurion University of the Negev, Beer-Sheva 8410501, Israel; nataliak@bgu.ac.il

**Keywords:** surrogacy, surrogate mothers, stigma, Russia, online support, coping, family norms

## Abstract

(1) Background: Gestational surrogacy is the most common type of surrogacy today. Although technologically well-developed and legal in many countries, it challenges and even contradicts the basic traditional concepts of family, motherhood, and gender roles. In the present study, we examined the types of stigma coping strategies surrogate mothers discussed in an online support group in post-Soviet Russia. (2) Method: We conducted a qualitative thematic analysis of 15,602 posts on a Russian-language online support group for surrogate mothers. (3) Findings: group members discussed four types of coping strategies: stigma internalization, stigma avoidance, group identification, and stigma challenging. Nevertheless, these strategies varied across the surrogate motherhood stages. Group members advised each other on specific strategies to use to cope with the state of discreditable (invisible) stigma (i.e., during the first few months of their pregnancies), with different strategies for when the pregnancies became visible and they risked becoming discredited people. Furthermore, group members disclosed that they used these strategies even when they returned to their previous family and work routines. Theoretically, our findings challenge Goffman’s classic theoretical dichotomy and coping research concerning discreditable (invisible) and discredited (visible) stigma. (4) Conclusion: Our findings indicate that surrogate mothers anticipate experiencing stigma and therefore plan for it by discussing potential coping strategies in the online group. Moreover, any intervention designed to cater to the needs of surrogate mothers must, therefore, take into consideration the social needs of their entire family.

## 1. Introduction

The current information age offers various online channels for obtaining social support to individuals who are coping with stigma. Online support groups are formed or facilitated through digital platforms, which provide a sense of space, shared practice, shared resources and support, shared identities, and interpersonal relationships [1]. Recent research points at different ways in which stigmatized individuals use digital platforms to actively cope with social stigma [2,3,4,5,6,7].

Drawing on stigma [8,9,10,11,12] and stigma online support group literature [1,7], in this study we investigated how surrogate mothers manage stigma in a Russian-language online support group for surrogacy. Commercial surrogacy—an arrangement supported by a legal agreement, whereby a woman (the surrogate mother) agrees to bear a child for another person or persons, who will become the child’s parent(s) after birth—is a legal arrangement in some U.S. states and in other countries including India, Russia, and Ukraine [13]. The increasing burden of infertility and the rising adoption of various surrogacy techniques are the factors driving this commercial market growth, which is expected to surpass USD 33.5 billion by 2027 [14]. Despite this growing market, surrogacy is stigmatized socially because it challenges—and even contradicts—basic traditional concepts of family, motherhood, and gender roles [15].

The context of surrogate motherhood in Russia is unique in the sense that, unlike Western and Asian surrogate mothers (SMs), post-Soviet SMs construct surrogacy as a kind of paid work [3,16]. Moreover, whereas in other countries, SMs are perceived as altruistic by helping childless couples [17,18], recent research about stigma among Russian surrogates revealed that Russian SMs experience the stigma of being labeled as bad mothers, bad wives, pathetic losers, and greedy women [3,19].

In this study we addressed the role of computer-mediated communication in coping with the stigma of surrogacy in Russia, thereby contributing to the study of stigma by applying extant offline stigma coping strategies to the online realm and by documenting the way SMs embrace or resist stigma through online communication. Unlike previous research that utilized surveys and interviews to examine stigma, in this study, we employed unobtrusive content analysis of messages posted on an online message board. The study also contributes to family, motherhood, and gender role literature by highlighting the ways in which local family norms influence the manifestation of different coping strategies in an online environment.

### 1.1. Strategies of Coping with the Stigma of Surrogacy

Generally, members of stigmatized groups have been depicted as passive victims of negative stereotypes, harmful attitudes, and discrimination. However, in the past two decades, stigma research has uncovered the distinct and varied ways that people cope with stigma [10,12,20]. In the present study, we focused on four coping strategies presented in stigma research: stigma internalization [11,12,21], stigma avoidance [22], stigma-challenging [12,20], and group identification [10]. A fifth stigma coping strategy, *distancing*, describes how stigmatized individuals can cognitively separate themselves from others who are similarly placed, and this has been examined in stigma research [23]. However, we decided not to include this strategy in our study as it cannot be traced through content analysis.

#### 1.1.1. Stigma Internalization

Stigma internalization, also referred to as self-stigma, is the process by which an external experience of stigmatization becomes part of one’s perception of self [24,25,26]. In the context of the current study, stigma internalization refers to individuals agreeing with broad cultural conceptions of surrogate motherhood and accepting those conceptions as truthful [12,20,21].

For example, a content analysis of Russian-speaking online support groups indicated that SMs are frequently told that they are bad mothers to their children and greedy for becoming surrogates [3]. Internalization of this stigma might involve the individual SM subsequently believing that she is indeed immoral. Another example of stigma internalization would be individuals withdrawing from social interactions because they think others will view them as bad mothers for becoming SMs. Thus, stigma internalization includes both emotional (i.e., feelings of alienation from others) and behavioral (i.e., withdrawal from social situations) components [25]. 

#### 1.1.2. Stigma Avoidance

The strategy of stigma avoidance involves the active avoidance of interpersonal interaction, in anticipation of devaluation [22]. The modified labeling theory [23,27] categorizes this strategy in two ways: (1) secrecy—concealing labeling information and keeping one’s reproductive status or choice a secret; and (2) withdrawal—dodging interactions with people who might be prejudiced or socializing primarily with others in a similar situation. This coping strategy may also involve what Goffman [8] conceptualized, and Yoshino [28] later developed, as “passing” and “covering”. In “passing”, individuals hide their stigmatizing characteristic so effectively that the “normal” are unaware of its existence. In “covering”, the stigmatizing attribute cannot be masked or is already known, yet the individuals mute it, so as to mitigate its delegitimizing impact on their social identity. Goffman [8] distinguishes passing from covering by noting that “passing” pertains to the visibility of the stigmatized attribute, whereas “covering” pertains to its obtrusiveness. Goffman [8] conceptualizes “covering” as a strategy to hide the stigma-causing attribute and thereby deflect attention from it. Strategy tactics include joking and attributing symptoms to something other than pregnancy, like weight gain or chronic illness. Thus, a discreditable person may attempt to pass for normal by concealing their condition from others. Sometimes, SMs will try to control the stigma by being highly selective about the individuals to whom they reveal their status and relying on these individuals to withhold this information from others. Nevertheless, concealing stigmatized attributes and avoiding disclosure can be a double-edged sword. As Charmaz [29] wrote, concealing a stigmatized characteristic, such as surrogate motherhood, may take an enormous amount of commitment, planning, and work.

Surrogate motherhood research has identified one way in which SMs conceal themselves. Saxena and colleagues [30] found that Indian surrogates make themselves invisible: they live in surrogacy hostels, engage in false claims of alternative employment, or even lie about the results of the pregnancy. Hence, Indian surrogates, by living in hostels for the whole duration of their pregnancies, manage to avoid the social stigma that might result in their being cast out of their communities.

#### 1.1.3. Stigma Challenging

At the opposite end of the spectrum of the previously mentioned stigma coping strategies are methods employed by those who oppose the widely accepted stereotypes concerning surrogate motherhood [12,20]. Surrogate mothers who challenge stigma directly will oppose what they consider to be biased beliefs, as well as challenge discriminatory behaviors against them or SMs in general. These women fight back rather than passively accepting the criticisms aimed at them. Challenging strategies include: (1) behaving in ways that contradict stereotypes; (2) educating others to reduce stereotypes; (3) confronting people who express prejudicial sentiments or discriminate against them; or (4) engaging in advocacy and activism [12,20,22].

#### 1.1.4. Group Identification

To reduce their stigma, stigmatized people are likely to search for others who share the same experience [8,12,31]. Consequently, members of stigmatized groups may cope with stigma by identifying more closely with the stigmatized group and approaching it for support [10]. In using group identification as a coping strategy, stigmatized individuals shift their social comparisons from their peers in the general population to similarly placed individuals. In addition, group identification is positively correlated with the self-esteem of members of stigmatized groups, suggesting that this strategy can effectively mitigate prejudices [32,33,34].

### 1.2. Russian Surrogate Motherhood Stigma

Researchers carrying out interviews or surveys with SMs have shown that surrogacy develops in a disapproving environment and as a somehow morally reprehensible experience [19,35] and that SMs tend to hide their involvement in surrogacy programs [36]. Even in the United States, where surrogacy has existed for 30 years, interviewees display some “cultural unease” [37]. As indicated previously, recent research into the stigma of surrogacy in Russia shows that surrogacy is associated with being a bad mother, a bad wife, a pathetic loser, and a greedy person.

Commercial surrogacy in Russia is minimally regulated—no governmental guidance exists regarding the contents of a surrogacy contract. The two main arrangement options for clients who enter a commercial surrogacy agreement are either by employing a commercial surrogacy agency that undertakes the selection of suitable surrogacy workers and all necessary communication and steps with clinics and lawyers or by taking matters into their own hands and choosing a direct arrangement [38]. Usually, the contract requires up to three embryo transfers (in case of failed transfers), the SM’s monthly payment for food and transport, compensation for medication and pregnancy-related expenses, and the final payment after successful delivery. Furthermore, it specifies “dos and don’ts” for the surrogacy worker at the discretion of the client parents. For example, prohibited behavior may include sexual intercourse, traveling, driving, or using public transport. Weis [38] documented, in her ethnographic research, the ways that local regulations limit the SMs’ decision-making rights. A Federal Law determines that the SM has the right to terminate the pregnancy like any other pregnant woman, up to the 12th week, or for medical reasons, at any point during the pregnancy. However, Weis notes that “the contract commonly stipulates these decision-making rights to lie with the client parents, pitching state law against contract law. Hence, if a surrogacy worker aborts without the client parents’ consent, the client parents can sue her and demand that she reimburses all incurred expenses” [38].

Local regulations also impact the SMs’ family life. Surrogacy regulations in Russia require that SMs must have between one and three healthy children of their own [39]. As such, by definition, in every SM contract in Russia, there are always children in the picture. Annually, 400–500 children are born via these services in Russia [39]. Nevertheless, surrogate motherhood is perceived as something that threatens family and motherhood core values and gender roles [15]. The Russian Orthodox Church has repeatedly called on the State Duma (the Russian Parliament) to ban surrogacy outright. The Church officially categorizes surrogacy as “morally reprehensible” [40]. In addition, public attitudes toward surrogate motherhood are negative. In 2013, the Russian Public Opinion Research Center survey found that only 16% of respondents rated surrogacy as entirely acceptable, whereas 26% of respondents perceived surrogacy as morally intolerable [19]. Thus, although surrogate motherhood is a legal procedure in Russia, the hostile attitudes of the public and the Church toward it construct the stigma against it.

Taking this local construction of surrogate motherhood stigma into account, it is essential to study how Russian SMs manage and neutralize the stigma associated with their “deviant” occupation [41,42,43]. Research into surrogate motherhood stigma in other countries has revealed the different ways in which SMs manage this stigma. For example, many SMs in the U.S. do not refer to their role as “work”, and distance themselves from those who view surrogacy as an act motivated by financial gain. Instead, they view surrogacy as a mission of gift-giving to childless “sisters” or couples [37]. Like U.S. surrogates, Indian surrogates also use this gift-mission-sisterhood rhetoric, but they view themselves as gift receivers: framing the opportunity to be a surrogate as a worthy gift from God [44]. At the same time, Berend [45], Imrie, and Jadva [46] revealed a more complex standing: although some SMs in the U.S. call surrogacy “giving the gift of life,” they describe their journeys using language that is associated with paid work. In another study, American, British, and Israeli surrogates approached surrogacy as both a contractual and gift-awarding relationship. They saw themselves as women who had an advantage (the ability to have healthy and enjoyable pregnancies and deliveries) that they were willing and excited to share with the intended family. Moreover, Israeli surrogates generally believe that “the payment does not eclipse the gift” because surrogacy creates a bond that is not about payment [17,47].

Recent research into stigma management among SMs from Russia highlights a different approach to surrogate motherhood. Weis’s [19,38] interviews with SMs revealed that keeping their occupation secret was often a strategic choice to avoid rejection, being belittled as a “breeder,” or discriminatory consequences for their children due to ignorance or someone else’s moral beliefs. A recent study of an online support group for Russian SMs revealed that the SMs cope with four different types of stigma: bad mothers, bad wives, pathetic losers, and greedy women [3]. It is this kind of surrogate motherhood stigma that seems to drive these women to online support groups as a source of support from others in a similar role.

### 1.3. Online Support from Others in a Similar Role

Social networks of others in a similar situation can be formed or facilitated through online communities, which provide a sense of space, shared practice, shared resources and support, shared identities, and interpersonal relationships [1]. In addition, computer-mediated communication offers a sense of immediacy through text and images rather than physical proximity and non-verbal cues. As a result, members meet fellow sufferers, escape their isolation in the physical world, and exchange support with others in similar circumstances.

Collectively, previous research suggests that online support groups offer advantages over other forms of support as they enable one to remain anonymous and overcome logistical obstacles [48,49,50]. Individuals who have a marginalized identity, and little offline social support, may turn to an online support group to compensate for the lack of those resources in their physical environment. These stigmatized individuals can reap the benefits of joining a group of others in similar situations: feeling less isolated and less different, disclosing a secret part of their lives, sharing experiences and learning from others, and receiving empathy.

Following research about SMs’ perceptions of their potentially stigmatized work, in this paper, we examined the coping strategies that SMs shared and discussed in online interactions on a Russian-language SM discussion board. As such, the current study illuminates the work of surrogacy, reveals the coping experience of SMs from Russia, and contributes to a growing body of literature on online support from other SMs.

## 2. Method

### 2.1. Inductive Thematic Analysis

We conducted an inductive thematic analysis to investigate how Russian SMs manage stigma through online communities [51,52]. Specifically, the thematic analysis examined online support group posts that included expressions of stigma coping strategies. We collected these posts from the Russian-speaking subsection “Donation and Surrogate Motherhood”, dedicated to surrogacy (https://www.ostrov-kenguru.ru/board/ accessed on 19 November 2020). This subsection represents an online community for Russian SMs, who shared and discussed their experiences with their peers. The content of this discussion board is accessible without website membership and the website archived all its forums so that data were explicitly public.

During the data collection period (January–March 2018), the entire support group had over 8400 members, 55 sections (sub-support groups), 6099 topics, and more than 1.5 million posts. In 2021, the entire online support group was shut down. The authors tried to contact the support group administrators to find out the background behind this move, but no available contact information was left. Informal communication with members of a different online support group for intended mothers in Russia revealed that the group was initially set up by a fertility clinic and evolved into a place (as this study shows) where SMs could voice their experience and even complain about their treatment by the funding clinic. Eventually, the clinic took it down.

Prior to data collection, we obtained institutional review board approval from the Ben-Gurion University of the Negev to conduct the study, which was exempt from further review as it relied on publicly accessible documents that do not require registration. Nonetheless, assuming that online support group members might perceive public digital spaces as private [53], and to mitigate the risk of exposing members’ information through an online search [54], we took the following steps: first, we assigned pseudonyms to the user names; second, we translated all posts published in Russian to English and edited the sentences; third, we removed all identifiable information, such as the names of people and cities that users mentioned. Lastly, we copied all the translated and edited quotes that are presented in this manuscript, searched for them on the Internet, and verified that none of them was traceable.

### 2.2. Data Analysis

To identify the themes emerging from the online discussion group, the second author (a native Russian speaker) first read all 213,505 posts from 278 discussion threads. Of these, 51 threads (15,602 posts) included discussions about SM stigma, descriptions of stigma-handling experiences, and advice on how to manage SM stigma. All 51 threads were retrieved, converted to MS Word, and translated into English. Both authors then imported them into Atlas Ti (qualitative analysis software) and read them in detail.

Our next step was to observe which stigma coping strategies were discussed in the online support group. To do so, we identified and coded all mentions of stigma coping strategies by analyzing units—words, sentences, or paragraph-long statements—that provided context for the ideas within each post (e.g., “keeping their attribute secret,” “avoiding social interaction,” or “telling a cover story”). Next, we narrowed down overlapping categories by discussing and comparing the 14 codes generated initially, agreeing on eight coding categories. Finally, we identified four main themes that embodied the way that support group participants discussed SM stigmas: stigma internalization, stigma avoidance, group identification, and stigma challenging. Once we identified these primary themes, we reread the posts, coded them, and placed them in a stigma coping strategy category. Out of the initial sample, 510 posts included expressions of stigma coping strategies. Within this group, 194 posts offered a combination of different stigma coping strategies. For example, we coded the following post as including both *stigma avoidance* and *group identification* strategies: “*Keep your mouth shut. Shut up and be quiet. Nobody, not at work, not relatives, not girlfriends. Especially girlfriends. If you need to speak out, come over and cry here*”.

We employed three steps during the analysis to enhance the study’s credibility and trustworthiness [55,56]. First, we used the equivalency test employed by Hamilton and Bowers [57] and searched for consistent information posted in different sections of the forum. Second, we discussed definitions of categories and themes, as well as interpretations of meaning. Third, we independently coded 300 posts for the presence of each category (0 = absent; 1 = present). Following the coding, we used Krippendorff’s alpha [58] to estimate inter-coder reliability. As Table 1 shows, for the final four categories, Krippendorff’s alpha coefficient varied from 0.76 to 0.89, indicating that coding reliability had reached acceptable levels.

## 3. Findings

### 3.1. Stigma Management Strategies

To uncover how online support-group members shared their efforts to handle stigma, potential dimensions of managing stigma were defined as mentioned above. As in many other cases of stigmatized individuals [8,10,21,22,24,59], these dimensions include: (1) the passive form of stigma internalization; (2) the active strategies of stigma avoidance—keeping their attribute secret, avoiding social interaction, “passing,” and “covering”; (3) group identification; and (4) stigma challenging in the form of education and confrontation. 

#### 3.1.1. Stigma Internalization (32 Posts)

Stigma internalization, the process by which an external experience of stigmatization becomes part of one’s perception of oneself [24,25], appeared in the posts mainly through the perception of being a “bad wife”. In their posts, the members described how their choice to be an SM caused suffering for their husbands. Sofia, an experienced SM (it was her third SM program), wrote:

“It’s easy to understand your husband’s frustrations. It’s hard for a man to accept that his wife is not pregnant with his child. You should not push him. You need to understand your husband, find the right way to approach him. This situation is harder for him than for you. It is also difficult for men to come to terms with the idea that they cannot (in their view) adequately provide for their families. They also care about relatives’ opinions ... husbands of SMs are often treated like pimps. After all, they earn money using their wives! He fears the condemnation that he, a healthy man, allows his wife to do such a thing instead of just working harder!”

Sofia was deeply concerned for her husband’s public and self-image due to her decision to become an SM. Her husband, guilty of nothing, was unfairly subjected to contempt. By becoming an SM, she was putting at risk her husband’s reputation, self-esteem, and social identity as a husband and a man.

Online support group participants shared their practices of coping with the internalized stigma of the “bad wife.” Some wrote about their efforts, in contrast, to become the ideal wife. For example, Annette, whose husband consented to her participation in the surrogacy program, wrote about how she tried to compensate for and reward him for his moral and physical “suffering”:

“My husband agreed on the condition that no one would know about the program. I put a lot of effort into making sure my participation in the program won’t hinder my husband. I cook his favorite dishes. I talk of how nice it’ll be to spend the money we get, try to support him somehow. I worry about my husband. We have not had normal intimacy for the last three months. I think this is very difficult for a man.”

Annette was grateful to her husband for consenting to her participation in the program. She considered it his sacrifice. Knowing that his friends and acquaintances would condemn him, he nevertheless allowed her to participate. As a result, she felt the need to compensate him: to cook something special for him, to concern herself with his sexual needs, to encourage him to dream about their future wealth. Thus, while internalizing the stigma of a bad wife, surrogates seem to try to use a few different approaches to improve their situation. They try to compensate for their husbands’ discomfort by becoming super-wives in other aspects. These strategies may help SMs deal with the internalized stigma of being a “bad wife”.

#### 3.1.2. Stigma Challenging (156 Posts)

Believing that stigma was based on ignorance, some SMs shared stories in the online support group about their efforts to challenge stigma by educating (69 posts) or confronting the people in their environments (87 posts). Education was manifested in the form of explaining the mechanism and social value of surrogate motherhood. Publishing posts with information about surrogacy in other online support groups, under pseudonyms, appeared to be a safe platform for this education strategy. Angela said:

“I recently tried to prove that being an SM is a noble cause at a mothers’ forum entitled: “Surrogate motherhood. Would you be able to?” I shared all the details, provided data, posted links to scientific articles. But it was me alone against hundreds. It was as if I could not be heard by them. Everyone responded by calling me a soulless prostitute, making money with my body, completely lacking any maternal instinct since I would sell my child for money. I argued and argued, and then I just got tired. It felt like I was trying to get water out of a stone. Everyone was stuck to their own opinions, and only a few supported me.”

Angela used the online support group features in order to educate: that is, she had access to a virtual community of young mothers, the anonymity to speak warmly and openly, and the ability to post links to relevant online resources. However, as she described, the stigmatizers resisted the information she provided. Additionally, some SMs used education strategies with those in their immediate environments, such as relatives. For example, Anastasia said:

“Why does the adult generation consider surrogate motherhood something terrible, scary, shameful???!!! At first, I thought it was because the majority simply did not understand how surrogacy works. People are very ignorant; they believe that a woman gives away her child or, even worse—she sells it. But it turned out that even if they are civilized people, and even if you describe to them the whole process, they still cannot accept it. Take, for example, my mom and dad. They are lovely people. But when I explained the process to them and said that I wanted to become an SM, I saw how my mother became nervous, and my father agreed to support me only on the condition that I would tell NOBODY about it. Even my brother! They still think that surrogate motherhood is terrible.”

As seen from the quotation, providing information about surrogacy did not change Anastasia’s parents’ attitude toward surrogacy. They still considered surrogate motherhood a problematic occupation, one that should be hidden from everyone, even family. Thus, the strategy of educating those in the SMs’ environment directly or via online support groups appeared to fail. The people in their environment resisted awareness; providing information about surrogacy did not overcome the stigma.

Some participants perceived their husbands as fragile: seeing them as being hurt as a result of their own (the SMs’) decision to become surrogates. They, therefore, considered it their duty to defend their husbands and confront the stigma on their own. Anna shared her opinion:

“I don’t give a damn about peoples’ attitudes, but my husband does care deeply. He worries, so I support him. If anyone says anything, I’ll react so badly that they’ll think twice about saying anything to us again. What’s important is that my husband and I both know that we earned that money and that another couple can have their little miracle, and these parents are happy. Soon the talk will dry up. Let them eat their poison.”

Anna perceived herself and her husband as partners, standing shoulder to shoulder to fight against the stigmatizing society. They perceived surrogate motherhood as a rewarding experience: an honest occupation that helped childless couples, and they would defend their honor.

#### 3.1.3. Stigma Avoidance (453 Posts)

Many members of the online support group wrote about how “dodging the stigma” worked for them. In their online communications, group members recommended to one another actively avoiding interpersonal interactions that were likely to make them feel devalued. Group members divulged that they withdrew from offline interactions with prejudiced people or people who inquired about their reproductive statuses. This strategy manifested in three ways: (1) secrecy—concealing labeling information and keeping one’s reproductive status a secret; (2) withdrawing from social interactions and familiar environments; (3) covering and passing—deflecting attention from the stigmatized attribute and using tactics such as joking and attributing symptoms to some other condition. The SMs moved between these three strategies according to how visible their pregnancies were.

#### 3.1.4. Secrecy (123 Posts)

When there was still little physical evidence of their pregnancies (and thus, their stigma was discreditable), a very widely employed strategy used by the SMs was controlling the stigmatizing information. They handled risks by dividing the world into two: a large group to whom they disclosed nothing, and a small group to whom they disclosed everything, and upon whose help they then relied. This division was challenging, as it was essential to find a balance between dodging stigmatizing social interactions and finding support. Sadly, this avoidance did indeed hinder receiving support from others. The SMs viewed self-disclosure as risky, potentially leading to rejection and banishment; therefore, secrecy was highly recommended, as Alice shared:

“It is better not to tell anyone. Neither friends nor relatives. You never know who in your surroundings is ignorant. Also, you will never know what people are actually thinking in their minds. They can say one thing but think in a completely different way (that often happens). They will kind of support you in words, but then it will come back to haunt you. Therefore, don’t try to explain anything, or prove anything—and tell no one! The risks are too high. I have no one. NO ONE knows that I am an SM.”

Alice had doubts about her relatives and friends and saw the disclosure of her surrogate motherhood status as a social threat. She was convinced that social sanctions would follow self-disclosure, even if she did not know exactly who would apply such sanctions. Moreover, she did not believe in the sincerity of people’s support. In her view, information about participation in the program would permanently weaken her position among friends and relatives.

Like Alice, many SMs did not even tell their parents, as Polina shared: “I believe that the fewer people know, the better. Explanations might not help. Therefore, I didn’t disclose it to my parents: I don’t want to take a risk and put my relationship with them to the test.” Polina admitted that even if she tried to educate her parents about surrogacy, they would not accept her choice to become an SM. She did not want to risk and disclose her occupation and consequently give up on receiving support. Polina’s fear, it should be said, was not without cause. For example, other online support group members shared that family members often denied them support after disclosing their SM status. For instance, Lenna wrote how her mother refused to host her during her pregnancy:

“I wanted my mom to know. I thought that I could stay with her during the late stages of my pregnancy and that she would help me a little with my son. I had no plan to hide from people because I’ve done nothing wrong. But my mother lives in a small town and she cares too much about people’s opinions. She says that they will remember this for the next 100 years. First, she seemed to support me, but then she said, ’Do not come to us when your belly is visible.’”

Lenna described her mother’s fear of the SM stigma spilling over onto the entire family. Her mother, fearing this stigma, refused to support her daughter once the pregnancy could be seen. Other SMs also needed to keep their occupation a secret for the sake of their loved ones, as Lolia noted:

“I don’t give a damn about the opinions of others. I would carry this pregnancy out in the open. But the children. I’m afraid for my children. They will be humiliated later, and I cannot harm them. And my husband, I feel sorry for him too. They’ll perceive him as guilty for allowing it. I agree that the fewer people know, the better.”

As disclosing their surrogacy even to their own parents was risky, and the chances of getting support were low, many SMs did not see the point in doing so. In addition, some SMs did not tell their relatives about their participation in the program, in order to spare them the stigma, as Daria divulged: “I decided not even to tell my mother. She does not need even more anxiety.” Thus, the SMs decided to narrow the circle of “sympathetic others” as much as possible, thereby reducing their social network and their chances of receiving much-needed support.

#### 3.1.5. Withdrawal (234 Posts)

In addition to secrecy, avoiding stigmatizing social interactions seemed an ideal option to many SMs. When their bellies were small, at the beginning of their pregnancies, this strategy did not require much effort. They stayed at home, and avoided going to public places or receiving guests, as Karin wrote:

“Well, you can stay at home for six months, not meet with anyone, not walk anywhere, and you can also live without theaters and shops. You can breathe the air late in the evening when it is dark and there are no people out on the street.”

Withdrawing from social interactions also meant hiding the pregnancy when visiting public places, such as the hospital, according to Karin:

“It is possible to hide it very well. In the winter I put on an extra-large coat and a massive scarf on top. If I went somewhere, I held a coat in my hands or some kind of bag. I also tried to stay out of view. For example, my husband drove up to the hospital entry gate and left quickly.”

However, withdrawing from stigmatizing interactions required more effort during the period when their pregnancies became visible. The online support group members shared stories about moving to other cities during this period, as Lola described:

“You don’t even worry about what people will say. People do not need to say anything. You go on a “business trip,” and you come back with money, and that’s it. Unfortunately, it is necessary to leave your children and husband for a long time. And then you have to search for work again.”

Or as Polina wrote:

“In the first program, my husband and I told our families and our children that we would be working in Italy for three months—the last trimester. We called them from ‘Italy’, and brought them ‘Italian’ gifts.”

As the quotes above suggest, the stigma coping strategy of avoiding social interactions had many limitations and disadvantages. It demanded finding a place to hide, from mid-pregnancy onward. It also required a long separation from the family, eventually also from the SM’s children. Moreover, this option did not allow SMs to continue to work in their regular jobs. Thus, avoiding the surrogacy stigma required a temporary interruption of social life and prevented the fulfillment of the women’s roles of wife, mother, and working woman.

#### 3.1.6. Covering (96 Posts)

Most online support group participants preferred to hide the fact of their participation in the SM program. However, those who could not leave their family or work had to make up stories to explain their bodies’ changes as the pregnancies progressed. To cope with the anticipated social judgment, the SMs shared three types of “cover stories” that helped them deflect the SM stigma. The first was pretending that they had gained weight. The second cover-up was pretending that they had been pregnant but lost the baby. The third strategy was to tell others that they were SMs but were doing so for a relative and not being paid for it. In the first two cover stories, the SM stigma was replaced by another stigma—being fat or losing the pregnancy. These two attributes were socially more acceptable. For example, Ksenia camouflaged her growing belly and explained her physical changes as weight gain:

“I was sometimes asked, ‘What’s up??? Are you waiting for the fourth one??!’ I always answered with a sarcastic tone—“Aha!!!—FIFTH!!!!” (Like, no way, we have enough!) Well, then I added with a sigh: “I need to eat less...”

Presenting herself as a weak-willed person, unable to resist overeating, seemed more acceptable to Ksenia than being an SM.

Making up a story about losing a baby was another possible strategy for SMs who could not hide their pregnancies. The online support group members advised one another on how to “playact”. First, one needed to express joy about the “future addition to the family” and discuss how the older children and the husband were waiting eagerly for the arrival of the baby. Then, after fulfilling their legal obligation and handing the child over to the biological parents, they suggested returning to work with a purposefully “sad face,” according to Annette:

“I was a surrogate twice while still holding down a job. I was entitled to maternity leave after each pregnancy but after giving birth to the surrogate baby, I went back to work. I informed management that the reason I was back at work was due to the loss of my child. I made a sad face and said that this topic was taboo for my family. I also told my acquaintances, ‘I don’t want to discuss it’... People usually do not ask further direct questions, and indirect questions might not be answered.”

Or, as Milana suggested:

“I learned not to talk much with strangers—again, not to attract attention—and if necessary, to tell lies. In my son’s kindergarten, they still communicate with me with a mournful half-whisper. The best strategy is to tell them: ‘Don’t want to talk about it, and don’t ask my son anything about it.’”

Hinting about the loss of the child discouraged information requests from those in the SMs’ social environments. For those who worked as SMs more than once, covering the stigma of being an SM by replacing it with the stigma of being a woman who miscarried was a successful strategy. Nevertheless, these performances were not easy for all SMs. For some, the need to lie and upset people made them feel uncomfortable. For example, Olga shared: “My neighbor was sincerely happy about our addition for the new year. When she recently called, I didn’t have the guts to lie and tell her I’d lost it.”

Several participants decided not to hide the fact that theirs were surrogate pregnancies. Instead, to avoid stigma, they preferred to keep silent about the commercial nature of the surrogacy. This covering-up strategy also seemed to be successful. Marta wrote:

“If anyone expresses interest in my pregnancy, I tell them right away, “My sister is infertile, and I’m helping her.” In the fertility clinic that hired me, I said that I was carrying for my sister. The attitude toward me was very kind. In the maternity hospital, too. Everyone thought I was having a baby for my sister (the biological mother and I even look alike a little).”

Marta chose to present her participation in the program as an altruistic act, as a sacrifice for the greater good of the family. This strategy allowed her to reveal her pregnancy; the people in her environment approved of surrogate motherhood as long as it was done altruistically and free of charge.

#### 3.1.7. Group Identity (63 Posts)

To reduce stigma and avoid discriminatory behavior, users of the online community identified more closely with surrogate mothers than with other mothers and approached the group for online support. Under these conditions, the community for many of its participants was a safe haven, as Faina admitted:

“My mother does not know, and neither do any of my relatives. So, I quenched my thirst for communication during both programs here. On the “Island,” you can rest your soul. You read familiar topics and stories and feel better because you do not feel weird or like a “special case.” It was only on the forum that I realized that we, the SMs, were actually doing a good thing, supporting a good cause!”

The role of the online support group in supporting the group identity strategy was vital, as the SMs felt they were understood by women who confronted the same challenges, as Galina wrote:

“In the first program, I didn’t know about the Island. This was a sad and lonely period. I was ashamed of being unmarried and pregnant. I thought I was the only single mother who was doing surrogacy. Now, after reading the posts I don’t feel like the ‘black sheep.’ No one can understand SMs like other SMs. Thanks for the support, my beloved Island.”

In some cases, identifying with group members took place even before joining a surrogacy program. Some members were women who were still considering becoming SMs. At first, they were only observers of the online group interaction (i.e., “lurkers”), as Nadia wrote:

“I have been reading the forum for a long time, and it’s so lovely to see how much kindness there is here. I decided to join you earlier to at least get a little support. Thank God there is a forum where you can get helpful information or vent your soul. In the real world, there is almost no one to talk to about such topics. So, add me to your ranks, please.”

Forming a group identity through the online support group was such an essential source of support for the members that some even felt it should be recommended as a source of support by the fertility clinics who hire them. Alice wrote:

“Every week there are more and more of us. It is a pity the clinics do not direct people to the online support group. So many girls go through the program alone. And here, they can always find advice and calm down.”

As the quotes from Nadia and Galina show, even passive participation, such as being an observer of the online support group, was helpful enough. The online community provided communication with people going through similar experiences and served as a digital haven. This community seemed to function as a place where SMs felt closer to the other anonymous participants than to their own relatives. They shared familiar topics and stories and created an online realm where being an SM was acknowledged as challenging but acceptable.

## 4. Discussion

In this study, we investigated the stigma coping strategies that SMs discussed in an online support group for surrogacy in post-Soviet Russia. In line with the stigma coping strategies literature [8,10,12,22,24,59], our findings revealed that surrogate motherhood discussion-board members discussed four types of stigma coping strategies in the online support group: internalization, avoidance (secrecy, withdrawal, covering and passing), stigma challenging, and group identity.

The communication among the SMs studied here reveals the efforts expended by the SMs in protecting what they perceive as a businesslike exchange. Although, in the post-Soviet context, SMs, professionals, and intended parents frame surrogacy work simply as a job [16], their immediate environment perceives it as a highly stigmatized occupation. By analyzing the SMs support group’s online communication, this study reveals the way they use the online support group to share and discuss strategies to cope with it. 

This study has several implications. Our findings expose an interesting dynamic around the use of stigma coping strategies throughout the pre- and post-natal stages. During the first few months of their pregnancies, when there were few physical signs of pregnancy, the online support group members shared and recommended coping with the discreditable/hidden stigma via a stigma avoidance strategy in the form of secrecy. However, when the pregnancy became physically visible, and the risk of becoming a discredited person appeared, the SMs recommended other strategies, such as withdrawing or covering and passing. Unlike people with, for instance, mental illness or epilepsy, who must consistently cope with stigmatizing attributes that are generally not visible, SMs must shift between strategies (as their stigmatizing attribute shifts from invisible to visible). Moreover, these coping strategies must change once again after the baby is handed over to the biological parents, and the SMs must return to their previous work and family routines. In this postnatal phase, the stigma coping strategy that they used was covering, making up stories of the loss of a child. As such, theoretically, this finding challenges Goffman’s [8] classic theoretical dichotomy and coping research concerning discreditable (invisible) and discredited (visible) stigma.

Empirically, our study highlights an interesting finding concerning surrogate motherhood stigma not only in post-Soviet Russia but also globally: it is a stigma that the SMs anticipate and can therefore plan for, by discussing potential coping strategies in the online group. In some cases, online coping with stigma is even practiced by women who are still only considering becoming SMs. These expectations lead to the use of the group as a springboard to discuss and prepare for the social ramifications that they fear. This process of preparing for an experience of stigma by engaging in an online group discussion may result from the social construction of surrogate motherhood as “dirty work” in post-Soviet Russia. Dirty work is viewed as a deviant occupation, as opposed to more legitimate or noble occupations and professions [60,61]. Our findings indicate that online group members are aware that their occupation may violate traditional family values and lead to moral sanctions. Therefore, they use the anonymous online discussion board to prepare for their anticipated upcoming experience of social shame and embarrassment. By forming a group identity and educating others about the SM experience, group members aim to challenge the view that surrogate motherhood violates the core values of motherhood. Sadly, as we reported earlier in this paper, the online community we studied no longer exists.

Practically, our finding regarding the SMs’ fear that the stigma will spill over to other family members emphasizes the need to provide SMs with a context of support and care that includes their entire family. Surrogacy regulations in Russia require that SMs must have between one and three healthy children of their own [39]. As such, by definition, in every SM contract in Russia, there are always children in the picture. Unlike those cases of family stigma constructed by culture or collective identities [8,62], we find here a stigma that, due to legal demands, spills over onto family members. Hence, the social ramifications of stigma include the SM herself, her children, and, in the event that she is married, her husband as well. Any intervention that is designed to cater to the needs of Russian SMs must therefore take into consideration the social needs of their entire family.

Although quantifying the use of online stigma coping strategies was not the goal of our study, it is nevertheless impossible to ignore the finding that most of the SM posts regarding coping strategies were devoted to stigma avoidance strategies. Specifically, the vast majority of the posts involved avoidance strategies (453), compared to 156 posts about challenging, 63 about identification, and 32 about internalization. Avoidance strategies were the most discussed topic and the most recommended of the strategies in the online community. Avoidance strategies were manifested in the ways that the community members taught each other how to conceal their pregnancies. Although our study method does not allow us to evaluate whether this strategy was indeed successful, our finding follows that of Lamba and colleagues [36], who compared the experience of Indian surrogate mothers and expectant mothers, both during their pregnancies and five months after giving birth, via questionnaires. This research found that surrogates hid their involvement in surrogacy to some extent and suffered from higher levels of depression compared to the comparison group during pregnancy and postpartum.

It is important to stress that stigma researchers [63] have found no evidence that coping orientations, such as secrecy, education, and withdrawal, buffer individuals from the harsh social ramifications of stigma. Individual coping orientations are unlikely to be effective and may even be harmful because they do not address the fundamental problem of fixed cultural concepts and stereotypes. The detrimental consequences of SMs hiding their pregnancies make clear that public health professionals must direct their efforts toward serving this vulnerable group of women. According to Link and colleagues, the best solutions are those that change societal conceptions of persons with the stigmatized condition or involve the collective action of people who are stigmatized.

Following Link and colleagues’ [63] advice, we hope that the SMs will find a new online space that will help shape a strong group identity, one that will eventually lead to collective action to help reduce the stigma regarding surrogacy. Nevertheless, the prevailing patriarchal-conservative ideology, combined with the weakness of civil society [64] in modern Russia, does not leave much room for hope that such manifestations of civic activism will take place. However, the public (albeit anonymous) disclosure of the details of these women’s experiences of stigma and discrimination, and their work on their collective identity, are in themselves social actions. Discussing the experience of stigma among SMs in a public and accessible online support group, such as that studied here, may be helpful for coping with surrogacy stigma. When Russian SMs are able to discuss their issues again in a public online support group, they will be one step closer to directly addressing society at large regarding the stigma toward SMs.

Practically, our study indicates that any international or global regulation of surrogacy needs to take the local moral frameworks towards surrogacy into account [16,65]. Thus, for example, if there existed an international policy framework or structure when planning any initiative toward surrogacy regulations [16,65,66,67], in addition to policies toward exploitation prevention and fair compensation of the SMs, the social consequences resulting from becoming an SM need to be taken into consideration. Furthermore, our findings indicate that beyond ensuring the legal status of SMs, surrogacy regulations must ensure the surrogacy industry (e.g., surrogacy agencies) honors its obligation to provide the SM with pre- and post-surrogacy mental health support. These support contexts can include health resources provided by surrogacy agencies for both SM and their family members and, as our study highlights in particular, support initiatives such as networks connecting other similar SMs.

## 5. Limitations and Future Research

The current study had a few limitations. First, it analyzed SMs’ discussion of stigma coping strategies under pseudonyms in a discussion board for SMs. This anonymity restricted us from examining the expression of stigma coping strategies per user as we had no information about users’ identities. In addition, this anonymity limited identifying how many distinct users used the online discussion board. Second, some posts included more than one single coping strategy, but we approached each strategy separately. Hence, the results do not indicate the richness of each message in terms of stigma coping strategies. Third, our study focused on the manifest content in the online posts, as with any type of content analysis. Therefore, we cannot indicate the users’ motivations or the “hidden meaning” underlying the posting and discussion of stigma coping strategies in the online support group. Lastly, we analyzed messages posted to a single online support group for SMs. The extent to which the observed pattern of stigma coping strategies is generalizable to other online groups has yet to be tested. Future research should seek to explore the categories of stigma coping strategies expressed, provided across multiple SM online support groups.

## Figures and Tables

**Table 1:** Overview of coding, definitions, and reliability.

Themes	Definitions	Examples	K-Alpha	Agreement
Internalization (32)	The process by which an external experience of stigmatization becomes part of one’s internal representation of oneself	*“I don’t know how my husband copes. What does he feel about my belly? I know I do it for our family, but I understand. I make him suffer.”*	0.76	78%
Avoidance— Secrecy (123)	Concealing labeling information and keeping one’s reproductive status or choice a secret	*“I do not say anything to friends; relatives do not know and will not find out. Even under torture, I will not tell any relatives about being an SM and how much I get paid. Only my husband and my doctor know. And you girls.”*	0.82	91.7%
Avoidance— Withdrawal (234)	Dodging or avoiding interactions with people who might be prejudiced, or socializing primarily with others who are in similar situations	*“After leaving my husband in the 2nd month of pregnancy, I moved to a biological mother spare apartment with my children. I talked with relatives on Skype—no one guessed anything: my belly was under the table, and my face “just got fatter.”*	0.85	91.6%
Avoidance— Passing and/or covering (96)	The SM’s pregnancy cannot be hidden. Yet the SM presents it as a sign of another, less stigmatizing, attribute.	*“I was pregnant in the winter. So yes, the neighbors noticed the growing belly. However, I told them that there was nothing to congratulate me on, that I was being treated with steroids. That’s why I was so swollen.”*	0.80	80%
Challenging— Education (69)	Educating others to reduce stereotypes	*“It was hard for me to persuade my husband. He was firmly against it. Like a woodpecker, I had the same conversation with him every day. I explained that this is a good thing, that this is helping people, that I will not sleep with anyone. I brought him brochures from the clinic, where everything is* *explained, and printed articles about* *surrogate motherhood for him. He was against it. He believed that we would be* *dishonored in the eyes of his relatives and would never get rid of the shame.”*	0.88	93.3%
Challenging— resistance (87)	Challenging stigma by resistance and confronting the stigmatizing environment	*“Everyone will be bothering me with the question—* *where is the child? Neighbors, mothers in the kindergarten, and relatives. I do not care. The main thing for me is to earn money for housing for my children and me, and I don’t care about others, even if I have to disclose it. Poker face and let the whole world wait.”*	0.79	80%
Group identity (63)	Identifying more closely with other SMs and approaching the group for online support	*“I use this support group every day. It is lovely to communicate with like-minded people, tell my thoughts, share the “secrets of surrogate motherhood” :-)). It is my shelter from my mad environment.”*	0.89	91.3%

## Data Availability

Data sharing not applicable.
